# Assessing the Effectiveness of Rehabilitation Interventions through the World Health Organization Disability Assessment Schedule 2.0 on Disability—A Systematic Review

**DOI:** 10.3390/jcm13051252

**Published:** 2024-02-22

**Authors:** Claudia-Gabriela Potcovaru, Teodor Salmen, Dragoș Bîgu, Miruna Ioana Săndulescu, Petruța Violeta Filip, Laura Sorina Diaconu, Corina Pop, Ileana Ciobanu, Delia Cinteză, Mihai Berteanu

**Affiliations:** 1 Doctoral School, University of Medicine and Pharmacy “Carol Davila”, 050474 Bucharest, Romania; claudia-gabriela.potcovaru@drd.umfcd.ro (C.-G.P.); teodor.salmen@drd.umfcd.ro (T.S.); miruna.sandulescu@drd.umfcd.ro (M.I.S.); 2 Department of Philosophy and Social and Human Sciences, Bucharest University of Economic Studies, Piata Romana. No. 6, District 1, 010374 Bucharest, Romania; dragos.bigu@man.ase.ro; 3 Department of Gastroenterology and Internal Medicine, University of Medicine and Pharmacy “Carol Davila”, 050474 Bucharest, Romania; petruta.filip@umfcd.ro (P.V.F.); sorina.diaconu@umfcd.ro (L.S.D.); cora.pop@umfcd.ro (C.P.); 4 Physical and Rehabilitation Medicine Department, Elias University Emergency Hospital, 011461 Bucharest, Romania; 5 Physical Medicine and Rehabilitation Department 9, University of Medicine and Pharmacy “Carol Davila”, 050474 Bucharest, Romania; mihai.berteanu@umfcd.ro

**Keywords:** disability, WHODAS 2.0, ICF, rehabilitation interventions

## Abstract

(1) Background: The World Health Organization Disability Assessment Schedule 2.0 (WHODAS 2.0) is a tool designed to measure disability in accordance with the International Classification of Functioning, Disability and Health. Measuring disability is becoming increasingly important due to its high prevalence, which continues to rise. Rehabilitation interventions can reduce disability and enhance functioning. (2) Objective: The present study aims to assess the impact of rehabilitation interventions on reducing disability, as measured by the WHODAS 2.0 questionnaire. It also seeks to identify which specific rehabilitation interventions are more effective and to explore other disability assessment questionnaires. (3) Methods: Following the Preferred Reporting Items for Systematic reviews and Meta-Analyses (PRISMA) methodology, we conducted a systematic review, with the protocol registered with the identifier CRD42023495309, focused on “WHODAS” and “rehabilitation” using PubMed and Web of Science electronic databases. (4) Results: We identified 18 articles from various regions encompassing patients with various health conditions, related to stroke, the cardiovascular system (cardiovascular disease, chronic heart failure), the pulmonary system (chronic obstructive pulmonary disease), the neurologic system (Parkinson’s disease, cerebral palsy, neurodegenerative disease), the musculoskeletal system (orthopaedic surgery), cancer, and chronic pain, and among frail elderly. These patients have received a wide range of rehabilitation interventions: from conventional therapy to virtual reality, robot-assisted arm training, exergaming, and telerehabilitation. (5) Discussion and Conclusions: A wide range of rehabilitation techniques can effectively improve disability with various comorbidities, offering numerous benefits. The WHODAS 2.0 questionnaire proves to be an efficient and reliable tool for measuring disability, and scores have a tendency to decrease after rehabilitation.

## 1. Introduction

The International Classification of Functioning, Disability and Health (ICF), which was endorsed by the World Health Organization (WHO) in 2001, represents a new paradigm in viewing disability from a biopsychosocial perspective, rather than just from that of a medical model. This offers a significant shift in the understanding and classification of disability, providing a holistic and integrative view [1]. Functioning and disability result from the interaction between health conditions and contextual factors (environmental and personal). More specifically, disability results when a given individual with an impairment in body function and structure interacts with the environment, resulting in a limitation on activity and restriction of participation.

**Figure 1:**
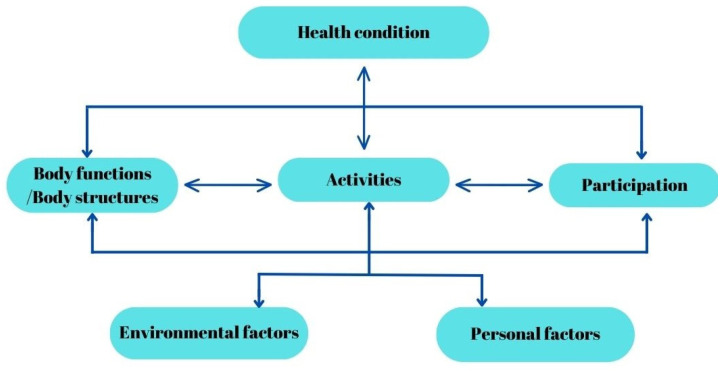
The ICF Model of Human Functioning and Disability.

The International Classification of Disease (ICD) offers an etiological framework for diseases, evolving from its first version in 1948 to the latest edition in 2022 [2]. To complement the ICD, the International Classification of Impairments, Disabilities, and Handicaps (ICIDH) was introduced in 1980, categorizing the consequences of diseases, thereby expanding the understanding and classification of health-related issues beyond the ICD’s scope [3]. The revision of the ICIDH was necessitated by its failure to incorporate the social model of disability, despite having established a tripartite system that differentiated between impairment, disability, and handicap [4]. Conversely, the ICF underscores how disability’s impact on an individual’s functioning varies from context to context, while maintaining neutrality towards the etiopathological aspects of disability. This approach aligns with ethical considerations in healthcare, such as informed consent, confidentiality, and respect for persons, offering a comprehensive perspective on disability that extends beyond traditional medical models [5]. This suggests that the ICF does not prioritize health statuses based only on medical hierarchies, so it helped to shift the focus from being only a disease classification, as seen in Figure 1 [1].

Around the world, approximately 1 billion individuals are living with disabilities, and this number is steadily increasing. This upward trend is influenced by factors such as higher life expectancy and a rising prevalence of chronic diseases. Within this diverse population, an estimated 110 to 190 million individuals encounter significant difficulties in performing everyday activities [6].

The assessment of disability is particularly challenging due to its context-dependent nature. Additionally, the perception of disability often varies between the patient’s subjective experience and the medical expert’s clinical observation, further complicating its measurement [7]. In the context of disability assessment, the “disability paradox” is an important consideration. It refers to the situation wherein individuals with significant disabilities often report a good or excellent quality of life (QoL), which differs from external perceptions of disability [8]. To provide a standardized method for measuring disability and health, the WHO developed and finalized in 2010 the WHO Disability Assessment Schedule 2.0 (WHODAS 2.0) [9]. It captures the level of functioning in six domains of activities in life: (1) cognition—understanding and communication (6 items); (2) mobility—moving and getting around (5 items); (3) self-care—hygiene, dressing, eating, and being alone (4 items); (4) getting along—interacting with other people (5 items); (5) life activities—domestic responsibilities, leisure, work, and school (8 items); (6) participation—joining in community activities (8 items). WHODAS 2.0 does not target a specific disease and can measure the impact of health or health-related interventions such as rehabilitation interventions. The questionnaire is available in multiple versions, including a long version with 36 questions and a short version with 12 questions, both assessing difficulties encountered in the last 30 days. The respondent is requested to rate the level of difficulty they encounter while performing a specified activity under normal conditions. The ratings they can choose from include the following: “none”, “mild”, “moderate”, “severe”, and “extreme or cannot do”. This evaluation should consider any reliance on supportive or assistive devices, as well as any aid received from caregivers [10]. WHODAS 2.0 has been translated into over 47 languages and dialects and has been used in over 27 areas of research, mainly in psychiatry (40%), but also in neurology and disability and rehabilitation (9%) [11].

Rehabilitation interventions necessitate a holistic strategy, reflecting the diverse array of diseases and conditions that result in various disabilities requiring rehabilitative care [12]. It is challenging to classify rehabilitation interventions because they often involve the simultaneous delivery of multiple treatments, integrated tasks challenging various body systems, and conversation-based approaches. The interventions are highly personalized and vary depending on individual patient needs and conditions [13]. In the realm of rehabilitation, interventions vary widely, encompassing several key aspects. Firstly, there are goal-oriented treatments tailored to alter the trajectory of a condition [14]. Secondly, a focus on reducing activity limitations is crucial, involving specific measures such as spasticity reduction [15]. Thirdly, interventions often centre around patient choice and participation, aiming to guide these choices through information dissemination or behavioural strategies [16]. Comprehensive assessments and goal setting play a pivotal role, entailing thorough evaluations for activity limitations and the establishment of both short-term and long-term objectives [17]. Equipping patients with the necessary skills and tools to lessen activity restrictions is another vital component, often involving the provision of assistive devices like wheelchairs. Additionally, modifying both physical and social contexts is essential to foster a supportive rehabilitation environment [18,19]. Lastly, the flexibility and multifaceted nature of these interventions underscore the need for a versatile approach that can simultaneously address a variety of challenges [20].

The objective of this article is to conduct a systematic review to investigate the impact of rehabilitation interventions on reducing disability, as measured by the WHODAS 2.0 questionnaire. Additionally, it aims to identify which specific rehabilitation interventions are more effective in reducing disability and to explore other disability assessment questionnaires that have been used alongside WHODAS 2.0 to assess disability.

## 2. Materials and Methods

A systematic review was performed according to the guidelines and recommendations from the Preferred Reporting Items for Systematic Reviews and Meta-Analysis (PRISMA) checklist. The protocol for this review has been registered with the identifier CRD42023495309.

### 2.1. Research Question and Search Strategy

An electronic search for relevant publications, performed using PubMed and Web of Science library databases, was conducted from 1 January 2013 to 18 December 2023. The following search strategy was used: “World Health Organization Disability Assessment Schedule” OR “WHODAS” OR “WHO-DAS” OR “WHO/DAS” OR “World Health Organization Disability Assessment Scale” (all fields) and “rehabilitation” OR “rehab” OR “rehabilitation program” OR “rehabilitation therapy” OR “rehabilitation intervention” (all fields). After this search, 589 articles were found (327 from PubMed and 262 from Web of Science). After applying filters for language (English), publication type (original articles), and date range (2013 to the date of the search), 211 articles remained. These articles underwent initial title screening, followed by an abstract review. Subsequently, 23 articles were fully read, resulting in a final selection of 18 articles. The research question was framed using the Population, Intervention, Comparison, and Outcome (PICO) method. The population was represented by patients with disabilities; the intervention by rehabilitation programs or a specific rehabilitation intervention; the comparison was against either a baseline measurement before the intervention or standard care without specific rehabilitation, or a control group with a conventional therapy; and the outcome was defined as the improvement in the WHODAS questionnaire scores, assessing changes in functional ability and disability levels post-intervention.

### 2.2. Inclusion Criteria

To be included in this review, studies had to meet the following publication criteria: (i) original full-text articles with randomized control and clinical trials; (ii) articles from the last ten years; (iii) articles published in English; (iv) on adult human populations.

### 2.3. Exclusion Criteria

Studies were excluded from the analysis if (i) they did not measure disability using WHODAS 2.0; (ii) they exclusively involved paediatric populations; (iii) the patients were presenting psychiatric ailments; (iv) the article did not provide a pre- and post-rehabilitation intervention assessment of disability using the WHODAS 2.0 tool; (v) the article involved cases where patients underwent surgery without a detailed explanation of the accompanying rehabilitation intervention; (vi) the article was solely a protocol study without presenting any results. Literature reviews, meta-analyses, case reports, and abstracts were also excluded from the selection, but they were used as a source for additional references.

### 2.4. Selection of Studies

**Figure 2:**
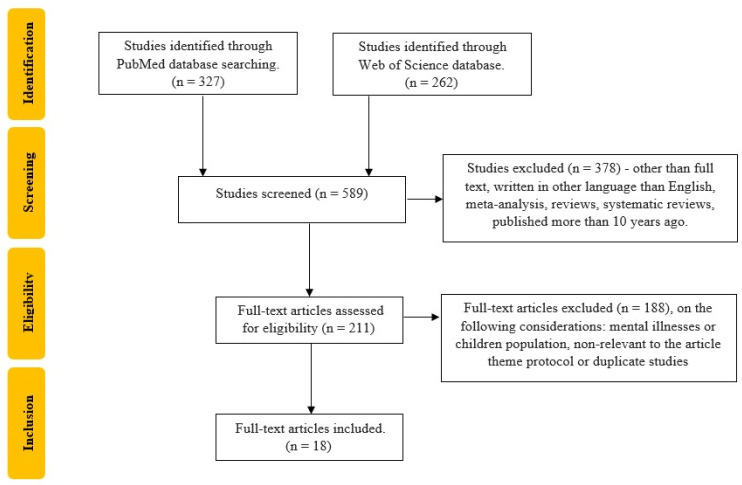
Flow chart of the study selection process according to PRISMA recommendations.

The study selection process was independently conducted by two reviewers (CGP, TS). Studies that did not meet the inclusion criteria and/or met the exclusion criteria were excluded. Each reviewer read the identified papers to ensure that all predefined criteria were met. The search was performed entirely independently and recorded by CGP and TS in separate databases and only compared at the end of the reviewing process to limit the selection bias. DC and MB resolved any disagreements that appeared, as seen in Figure 2.

### 2.5. Data Extraction

Two authors used a self-made data extraction table to individually evaluate and extract the following data for each included literature: the first author, year of publication, geographic region, study design, sample size, average age of participants, condition that led to disability, rehabilitation intervention details, WHODAS 2.0 version used (12-item, 36-item), values of WHODAS 2.0 before and after the intervention, and other evaluation tools. Any differences of opinion were settled through discussion or consultation with a third author.

### 2.6. Risk of Bias Assessment

The quality of the studies was assessed independently by two reviewers, with the Newcastle–Ottawa Scale (NOS) and the results are summarized in Table 1.

### 2.7. Strategy for Data Synthesis

The narrative synthesis of the study findings focuses on the type of rehabilitation strategy and the improvement in disability. For each rehabilitation intervention, at least 2 studies are included. In addressing the differences in methodologies during the synthesis process, we employed several strategies. Firstly, we carefully documented and categorized the variations observed across the included studies. Secondly, we conducted a thorough quality assessment of each study to ascertain the reliability and validity of their methodologies. Given the expected heterogeneity of the studies (in terms of design, quality, screening methods, interventions, and outcomes), a narrative synthesis approach was employed. This involved using text and tables to offer a descriptive summary and explanation of the characteristics and findings of each study.

## 3. Results

This systematic review encompasses 18 studies published between 2014 and 2023. Table 2 summarize a part of the information extracted from the selected studies, as described below. These studies span various regions including Europe (Czech Republic, Italy, Norway, Sweden, Spain, Finland), North America (Canada, United States), South America (Brazil), and Asia (Japan, Taiwan, China, Vietnam, Iran). The WHODAS 2.0 questionnaire was used to measure disability among various health conditions, such as ischemic and haemorrhagic stroke, cardiovascular disease (CVD), chronic heart failure (CHF), chronic obstructive pulmonary disease (COPD), Parkinson’s disease (PD), Gulf War illness, chronic pain, traumatic injury, hip arthroplasty, cancer, neuromuscular diseases (NDs), innate disability, and others; with sample sizes ranging from 4 to 3506 participants. Regarding the WHODAS 2.0 type of questionnaire, 10 studies used the 36-item version (55.5%), 6 studies the 12-item version (33.3%), and 2 studies employed both versions (11.1%). The included studies in the review were all prospective, encompassing a variety of research designs such as randomized controlled trials (RCTs), quasi-RCTs, and longitudinal, cross-sectional, and mixed-method studies. The participant demographics across these studies varied significantly, with some focusing on older populations, others on broader age ranges, and a few not reporting the mean age. This diversity in study designs and participant demographics adds to the comprehensiveness of the review’s findings.

Table 3 summarizes the rehabilitation interventions and their effectiveness, as measured using the WHODAS 2.0 questionnaire at various stages including baseline, post-intervention, and follow-up. In addition to the WHODAS 2.0 assessment, other tools were utilized for the selection and complementary evaluation of patients.

The rehabilitation interventions in this review showcase a remarkable range of approaches, as seen in Table 3. Virtual reality (VR) with conventional rehabilitation therapy (CnvT), compared to CnvT plus additional therapies like iodine–bromine baths and oxygen therapy [21]; remote versus hospital-based cardiac rehabilitation (CR) [22]; the SIDERA^B program, a comprehensive telerehabilitation system that includes various forms of training such as endurance, resistance, and neuromotor training, tailored for home-based management of chronic conditions [23]; problem-solving treatment (PST) and health education (HE) via telephone [25]; use of existing healthcare system services in Taiwan [26]; robot-assisted arm training (RAT) compared to CnvT [27]; low-frequency sinusoidal sound vibration and self-care treatment [28]; diet quality modification [29]; the CaRE@Home program, with aerobic, resistance, and flexibility training, plus e-learning and health coaching [30]; functional training, bicycle exercises, and exergaming [32]; home-based resistance exercises [33]; dance classes versus kinesiotherapy [35]; powered mobility devices (PMDs); day care service with a range of medical and rehabilitative services [36]; an online psychosocial program [38]. These results reflect a broad spectrum of rehabilitation strategies and their varied impacts on different patient groups and conditions.

WHODAS 2.0 scores change over time, indicating the impact of interventions. For example, scores generally decrease (improve) after interventions and over follow-up periods, as seen in Table 3. In some cases, the scores between the intervention group (IG) and control group (CG) are similar [21], whereas in others, there are significant differences [22,24,26,34]. The effectiveness of rehabilitation interventions also varies depending on the time elapsed since the intervention [25,30,32,36,37,38], and also with the rehabilitation intervention used [33], as seen in Table 3.

Additionally, Table 3 includes various evaluation tools used alongside WHODAS 2.0 in the included studies. These tools provide a thorough assessment of different health outcomes, evaluating patients’ physical, cognitive, and psychosocial functioning, as well as their QoL, in response to diverse rehabilitation interventions, which include the following:

- Cognitive assessments: MMSE (minimal mental state examination), MoCA (Montreal Cognitive Assessment) [21,27];
- Functional independence measures: BI (Barthel Index), EBI (Extended BI), FAC (Functional Ambulatory Category), FIM (functional independence measure), and ADL (activities of daily living) [21,24,27,34,35];
- Balance and mobility tests: BBS (Berg Balance Scale), 6MWT (6 min walk test), 10MWT (10 m walk test), FTSST (Five-Time Sit-to-Stand Test), and TUG (Timed Up and Go) [21,32,33];
- QoL: WHOQOL-BREF (shortened World Health Organization QoL scale), SF-36 (Self-administered Short Form Health Survey), EQ-VAS (EuroQol Visual Analogue Scale), and SIP (Sickness Impact Profile) [22,31,33,37,38];
- Disease-specific scales: PDQ-39 (Parkinson’s Disease Questionnaire-39) for PD [32];
- Usability and acceptance scales: SUS (System Usability Scale), TAM3 (Technology Acceptance Model 3), and SUTAQ (Service User Technology Acceptance Model) [23];
- Physical activity and diet measures: Physical Activity Questionnaire, DASH-Q (Dietary Approaches to Stop Hypertension Quality questionnaire), IPAQ-SF (International Physical Activity Questionnaire, short version) [29];
- Other assessments: VAS (Visual Analogue Scale) for pain, Beck’s Depression Inventory-II, Hospital Anxiety and Depression Scale for mental health [28,31].

## 4. Discussion

We investigated how diverse rehabilitation interventions can effectively reduce disability as assessed by the WHODAS 2.0 questionnaire in various health conditions including stroke, CVD, CHF, PD, COPD, knee or hip arthroplasty, Gulf War illness, chronic pain associated with potential depressive and anxious symptoms, cancer and other chronic diseases in older people with or without frailty, and innate NDs. Rehabilitation interventions are diverse, ranging from CnvT, VR, and RAT to remote rehabilitation with telerehabilitation programs, psychosocial interventions, and the use of mobility devices (PMDs).

The diversity of the countries involved in this systematic review, including representants from almost all the continents, including the Czech Republic, Japan, Italy, the United States, Taiwan, China, Finland, Vietnam, Canada, Norway, Brazil, Iran, and Spain, highlights the cross-cultural applicability and relevance of rehabilitation interventions in reducing disability as measured by WHODAS 2.0. This aligns with the global utilisation of the questionnaire, which, by the end of 2015, had been administered in nearly 100 countries and translated into almost 50 languages and dialects [9]. The fields of geriatrics, neurology, disability, and rehabilitation research show a significant interest in WHODAS 2.0, accounting for 23.9% of the research, although this is less than the interest of psychiatry, which constitutes 40% of the research focus [11]. This geographical diversity incorporates a wide range of healthcare systems and rehabilitation practices, offering a comprehensive global perspective on effective strategies to diminish disability across various conditions. There are also disparities among healthcare systems in understanding disability [39]. The vast diversity of conditions leading to disability, coupled with significant cultural differences, highlights the necessity for individualized rehabilitation treatment for each patient [40]. Incorporating patient-reported outcomes and perspectives into rehabilitation research could offer crucial insight into how patients perceive and accept various interventions. This approach can help assess the real-world impact of rehabilitation strategies from the viewpoint of those directly affected, leading to more patient-centred and effective care solutions. Considering the wide-ranging impact of diseases on disability and the diverse array of comorbidities and individual patient characteristics, it becomes evident that creating a universally applicable rehabilitation program is challenging. The heterogeneity among patients and the unique responses to therapy further complicate this task. The significant variability observed across studies makes it impractical to conduct a meta-analysis. However, through a systematic review, we can comprehensively gather and analyse the diverse spectrum of rehabilitation strategies and their outcomes. This approach allows for a nuanced exploration of the available evidence, offering valuable insights into the effectiveness and applicability of different rehabilitation interventions.

The presence of methodological diversity highlights the complex nature of the research in rehabilitation strategies. While methodological differences may introduce variability in the results, they also enrich the depth of evidence available, offering insights into the effectiveness of different approaches across various contexts. By acknowledging and addressing methodological variances, we strive to enhance the reliability and generalizability of our study findings, thereby facilitating informed decision-making in clinical practice and policy formulation. Sewell et al. [41]. found that individually targeted exercises for COPD patients did not show significant differences compared to generic exercises, while Jen et al. [26]. demonstrated that the Taiwanese healthcare system significantly improved disability outcomes in stroke patients over a four-year period [26,41]. The move towards personalized treatment in healthcare, including for conditions like COPD, CVD, and stroke, reflects an evolving understanding of patient care. Despite studies showing no significant differences in outcomes between individualized and generic exercise programs in terms of disability reduction, the trend is leaning towards customization. This approach considers individual patient needs, preferences, and specific health conditions, aiming to optimize treatment effectiveness and patient satisfaction by tailoring interventions to each person’s unique situation. Additionally, active patient involvement alongside a multidisciplinary systems medicine approach is crucial to provide predictive and preventive healthcare in the management of COPD [42,43]. Taiwan’s healthcare system is unique due to its National Health Insurance model, providing universal coverage and emphasizing efficient use of technology. In comparison, other countries might use multi-payer systems, like the United States, or government-funded models, like the UK’s National Health Service. Taiwan stands out for its high satisfaction rates, cost-effectiveness, and innovative use of smart health cards, which streamline patient information and services. This contrasts with systems facing challenges like higher costs, unequal access, or less integration of health technologies [44].

Regarding the two versions of the WHODAS 2.0 questionnaire (12-item and 36-item), the data reported in the literature show a more homogeneous use, with 29.5% using the 12-item and 30% using the 36-item version, while other studies do not report the questionnaire type used. However, our research found usage rates of 33.3% and 55.5%, respectively [11]. Both the 12-item and 36-item versions of WHODAS 2.0 are recognized for their substantial validity and reliability. The choice between them depends on the researcher’s preference and the specific requirements of the study [11].

Information on the administration methods, respectively, interviewer-administered, self-administered, and proxy-administered, of the WHODAS 2.0 form is scarce; only one study indicated that it was conducted through interviews, another that it used a self-administered approach, and a third one documented both self-administered and proxy-administered methods. Kilkki et al. [24] found that in stroke survivors, proxies perceived the disabilities to be more severe than the survivors’ own perceptions. This aligns with the “disability paradox”, wherein individuals with significant disabilities often report a good or excellent QoL, contrasting with external perceptions of their condition [8]. Fellinghauer et al. [45] discussed the “disability paradox,” noting that a significant number of individuals with impairments report good health and QoL, influenced by contextual factors such as socioeconomic determinants [45]. There is a potential for bias in disability evaluation, as there is a tendency for reported disability to decrease over time, as we can see in Table 3. This raises a crucial question: is the reduction in WHODAS 2.0 scores a result of genuine improvement from rehabilitation interventions, or merely an adaptation to disability influenced by contextual factors? Distinguishing between actual functional improvement and patient adaptation can be extremely challenging. This complexity highlights the importance of nuanced evaluation methods to accurately assess the effectiveness of rehabilitation interventions [46,47]. Self-reported limitations in activities can be influenced by cultural and reporting biases. In some cultures, not being able to perform basic self-care might not be seen as a limitation due to societal norms or scarcity of resources like water. For example, in a community where running water is not the social norm, and people are not accustomed to having constant access to water, they may not consider the lack of ability to utilize it as a limitation. In contrast, for someone who is used to continuous access to water, its absence could be perceived as a limitation [48]. Additionally, cognitive impairments can affect individuals’ abilities to prioritize tasks, potentially skewing self-reporting in assessments [49]. Age is another significant factor that can influence the WHODAS 2.0 scores; in a study of patients admitted to an intensive care unit (ICU), there were statistically significant differences in the WHODAS 2.0 scores between patients more than 65 years old and those under 65 before admission to the ICU [50].

Galli et al. [34] compares WHODAS 2.0 and the MBI in assessing disability and recovery in orthopaedic rehabilitation. They concluded that both tools should be used together for a comprehensive assessment, as WHODAS 2.0 incorporates the patient’s perspective on disability, while the MBI provides a more objective measure of functional independence. At baseline, the MBI had an average disability score of 55.06 ± 1.380. WHODAS 2.0 had an average disability score of 62.72 ± 1.010, indicating severe difficulty. Interestingly, patients reported a lower perception of their disability when using WHODAS 2.0, compared to the clinicians’ assessments of their disability using the MBI. At discharge, the data revealed the following scores: WHODAS 2.0 had an average score of 84.35 ± 1.300, and the ADL BI had an average score of 93.19 ± 1.210. These results suggested a slight residual disability at the discharge phase. During the follow-up period, the perceived recovery rate, based on WHODAS 2.0, increased by +36.39%, rising from 62.72 to 84.35. In contrast, the recovery rate measured using the ADL BI showed a more substantial increase of +77.69%, going from 55.06 to 93.19. These findings highlight the difference in perception between patients and clinicians when assessing disability and recovery. While WHODAS 2.0 indicated a lower disability level, the ADL BI suggested a higher level of recovery. This emphasizes the importance of considering both perspectives for a comprehensive evaluation of patients’ well-being [51].

Practically any health condition has the potential to impair functioning. The impact often varies greatly depending on the disease, age, and the person’s perception and adaptation to their situation, highlighting the high variety and subjective nature of disability and the importance of considering psychological and social factors in the rehabilitation process alongside a personalized treatment [52]. Campbell et al. [28] highlighted that pain could lead to disability, even in middle-aged individuals, and emphasized that rehabilitation interventions like vibroacoustic therapy (VAT) can improve the outcomes of medical treatments for pain [28]. McAndrew et al. [25] used PST and HE and observed pain reduction and a significant disability reduction (*p* = 0.04) within 6 months among individuals with Gulf War illnesses [25]. Other methods for alleviating pain include electrotherapy techniques such as Transcutaneous Electrical Nerve Stimulation (TENS) and High-Intensity Laser Therapy (HILT), which are well documented and have been studied for their effectiveness [53]. This suggests the importance of integrating rehabilitation strategies to manage pain-related disabilities effectively [54].

Five articles assessed improvements in disability through various rehabilitation interventions in individuals who have experienced stroke. Dabrowska et al. [21] observed that the application of VR did not result in significantly different outcomes regarding WHODAS 2.0 results compared to CnvT after 4 weeks [21]. In contrast to Dabrowska et al.’s [21] findings, Chen et al. [27]. discovered that RAT significantly improved WHODAS 2.0 scores (*p* = 0.01) when compared to CnvT [27]. Other studies have found that combining RAT with CnvT in the early rehabilitation phase after a stroke is more effective than CnvT alone. This approach improves gross manual dexterity, upper-limb functionality during tasks, and the social participation of patients. However, these studies did not measure disability outcomes using the WHODAS 2.0 questionnaire [55]. Levin et al. [56] also found a modest advantage of VR over CnvT, and support further investigation [17,56]. However, Kilkki et al. [24] found that having access to rehabilitation like physiotherapy, speech therapy, occupational therapy, and neuropsychological counselling significantly improved WHODAS 2.0 scores at 9 to 50 months after rehabilitation (*p* = 0.004) [24]. Jen et al. [26] reported a significant improvement in stroke patients in WHODAS 2.0 scores (*p* < 0.05) following 4 years of rehabilitation within the Taiwanese healthcare system. Nguyen et al. [29] found that stroke patients with comorbidities had significantly higher disability scores compared to those without comorbid conditions, as indicated by a regression coefficient (B) of 8.24 and a 95% confidence interval (CI) of 6.66–9.83 (*p* < 0.001). He also reported that physical activity (PA) diminished disability in those with comorbid conditions. Individuals in the second and third tertiles of PA exhibited disability scores that were 4.65 and 5.48 points lower, respectively, than those in the lowest tertile, indicating a significant reduction in disability with increased PA [29]. Additionally, PA has a beneficial effect on a number of cardiovascular and metabolic risk variables that make up or are associated with metabolic syndrome, a significant concomitant risk factor for stroke [57,58].

Fukuta et al. [22] discovered that remote CR led to a significant reduction in WHODAS 2.0 scores, thereby improving disability, in comparison to the hospital-based CR group (ΔWHODAS 2.0-J score: −8.56 ± 14.2 versus 2.14 ± 7.6; *p* < 0.01) after a 12-week period. Their findings support the effectiveness of remote CR as a viable option for treating stable patients who are unable to attend hospitals [22]. The pandemic caused by the SARS-CoV-2 virus has compelled us to find innovative solutions for remote communication and healthcare delivery. In this context, telerehabilitation programs have become essential and are demonstrating promising results. These are particularly beneficial for stable patients as they not only provide a structured program but also offer regular motivational support. As a result, these programs can motivate the patient and can lead to achieving comparable or even superior results in some cases, compared to traditional methods of rehabilitation. Telerehabilitation programs have gained momentum across various medical conditions, including stroke, CVD, PD, pulmonary disease, and orthopaedic surgery [59,60,61]. Rossetto et al. [23] developed a telerehabilitation system called SIDERA^B, an acronym for “System for Integrative Digital Education and Remote Assistance in Health”. This system delivered telerehabilitation activities through multimedia digital content and telemonitoring of vital parameters using technological devices for home-based management of chronic conditions.

The program for chronic heart failure (CHF) lasted for 3 months, while the programs for PD and COPD lasted for 4 months. These programs included endurance training, resistance training, and neuromotor training. The level of disability is associated with the ease of using telerehabilitation. The WHODAS 2.0 total score exhibited a negative correlation, with a rho value of −0.218 (*p* = 0.021). Higher disability levels are linked to a less straightforward perception of ease of use.

Martinez et al. [38] reported that teleassistance improves QoL in people with NDs. The analysis of the WHODAS 2.0 questionnaire revealed that the experimental group, which participated in a 3-month online psychosocial program based on CBT principles (including psychoeducation, coping strategies, emotional support, and stress reduction techniques like guided imagery, cognitive restructuring, and problem-solving strategies), exhibited significant improvements in various domains, with medium-to-large effect sizes (ranging from r = −0.42 to r = −0.79), such as “Understanding and communicating”, “Getting along with people”, “Life activities”, “Participation in society”, “Total WHODAS working”, and “Total WHODAS not working”, whereas the control group showed limited changes, except for the “Life activities” domain, which increased from pre-test to post-test (z = −1.960, r = −0.43, *p* ≤ 0.05) [38].

Telerehabilitation aims to reduce disability within a specific category of patients. It is crucial to carefully select patients who can benefit the most from telerehabilitation because a high level of disability and limited familiarity with electronic devices can be a disadvantage. This is especially true for elderly patients who may also have visual impairments and for patients with significant disabilities who may have associated cognitive impairments. While research in this area has shown promise, it is essential to continue evaluating and refining these technologies to ensure their clinical quality and effectiveness compared to traditional face-to-face rehabilitation [62,63].

In contrast to telerehabilitation, the study by Teixeira-Machado et al. [35] highlights the distinctive advantages of engaging young individuals with cerebral palsy (CP) in dance classes informed by Feldenkrais, Horton, Graham, and Laban/Bartenieff concepts. These classes offered a hands-on, interactive experience that integrated physical movement with expressive elements, thereby enhancing motor skills, social interaction, and emotional well-being through the artistic and rhythmic aspects of dance. Conducted over 24 sessions (1 h, twice a week, for 3 months), the study found significant improvements in various domains including independence function, mobility, communication, psychosocial adjustments, and cognitive function in the dance group compared to the control group undergoing traditional kinesiotherapy, which included Bobath and Kabat methods, Frenkel exercises, and proprioception exercises. WHODAS 2.0 showed significant improvement in the dance classes rehabilitation group after 24 sessions, with the most significant improvements observed in the “participation” section, attributed to psychosocial adjustments (*p* = 0.04) [35].

Research has shown that cancer survivors face various functional challenges due to the disease and treatment side effects. If left unaddressed, these issues can result in long-term functional decline, psychological distress, and reduced QoL. With an increasing number of elderly cancer survivors, there is a demand for improved access to rehabilitation services. Telerehabilitation, utilizing technology to enhance communication and accessibility, offers a promising solution to address this need and improve outcomes for cancer patients [64].

The CaRE@Home study by MacDonald et al. [30] focused on an 8-week online multidimensional cancer rehabilitation program, incorporating 150 min per week of moderate-intensity aerobic exercise, resistance training, and flexibility exercises, supplemented by e-learning modules and health coaching calls. This program demonstrated significant reductions in disability scores and improvements in physical activity levels, work productivity, and physical performance measures among participants [30]. In the Hustoft et al. [31] study, patients with neoplasms experienced a notable reduction in WHODAS 2.0 scores, indicating decreased disability, after a 3-week rehabilitation process. The scores improved from an average of 28.6 at baseline to 24.1 after 1 year. The study found a significant association between higher Rehabilitation Complexity Scale (RCS) communication scores and improved health outcomes in the neoplasm patient group, as reflected in WHODAS 2.0 scores (b = −20.66, 95% CI = −37.05, −4.28, *p* = 0.013). This suggests that effective communication within rehabilitation can positively impact health for these patients. However, the study did not find a significant relationship between Nurse Care Quality-N (NCQ-N) scores and changes in WHODAS 2.0 scores when analysing by referral diagnosis groups, indicating that the quality of nursing care might not directly influence these outcomes in the same way [31]. Comparing these two approaches, CaRE@Home offers a structured, multidisciplinary online intervention with a clear focus on physical rehabilitation and self-management for cancer patients, showing quantifiable improvements in disability and physical function. The study by Hustoft et al. [31] presents a more generalized view of rehabilitation care’s impact, emphasizing the role of team collaboration and continuity but with less focus on specific intervention outcomes and their direct impact on disability reduction.

Three studies, performed by Ferraz et al. [32], Shahbazi et al. [36], and Petterson et al. [37], focused on interventions for improving the well-being and functionality of elderly individuals.

Ferraz et al. [32] recruited 62 elderly individuals randomly divided them into three groups: a functional group (G1), a bicycle exercise group (G2), and an exergaming group (G3). All groups demonstrated significant improvements in the following measures: 6MWT, sitting–rising test (SRT), and WHODAS 2.0. Only G3 showed a significant improvement in gait speed during the 10MWT. G1 and G3 reported improved perception of QoL based on EuroQol-5D and PDQ-39. G2 and G3 achieved significant reductions in abdominal circumference. Regarding disability measured by WHODAS 2.0, all three intervention groups showed statistically significant improvements in the measured outcome over the 8-week period. The *p*-values confirm the significance of these improvements, with the functional training group having the lowest, *p* = 0.018, followed by bicycle exercise, *p* = 0.019, and exergaming, *p* = 0.041 [32], as seen in Table 3. Exergaming and bicycle exercises also demonstrated positive outcomes in terms of working memory when compared to the control group. However, they did not lead to significant reductions in blood pressure. Furthermore, video games that require body movements have the potential to improve balance and muscle strength, especially if combined with resistance training in frail elderly patients [65]. Exergaming stands out as an intriguing and promising exercise approach [66,67].

Shahbazi et al. [36] show that a rehabilitation service package had a significant positive effect on reducing disability scores in various domains among older people in the case group compared to the control group over a 6-month period. The most significant improvements were observed in the domains of “getting along with people” and “getting around”. At the beginning of the study, disability mean scores were, in the case group, 22.6 ± 11.2, and in the control group, 22.0 ± 11.5. The highest disability mean scores were for “getting around” 35.5 ± 20.8 and for “life activity” 33.5 ± 22.7. After 6 months, the disability mean score in the case group was 17.4 ± 8.9, while in the control group it was 25.8 ± 10.8 [36], as seen in Table 3. This indicates that the provision of rehabilitation services within the primary healthcare system in Iran is linked to a wide range of health and social advantages. When compared to Taiwan, both countries have observed benefits and a reduction in disability over time [26,68]. Cultural and contextual factors play a crucial role in influencing the effectiveness and applicability of rehabilitation interventions across different populations. The observation that both countries have experienced similar benefits and reductions in disability over time underscores the importance of recognizing variations in healthcare infrastructure, societal attitudes towards disability, and access to resources across different countries and cultures. These factors can significantly influence the implementation and effectiveness of rehabilitation programs. To design interventions that are specifically tailored to the needs and contexts of a diverse population, ultimately maximizing their effectiveness and applicability, it is imperative that these cultural and contextual factors be understood and addressed.

Petterson et al. [37] provide evidence that the use of powered scooters (PSs) can significantly enhance the lives of older individuals by improving their ability to perform daily activities, participate in various roles, and experience an overall better QoL. While there was statistical significance (*p* = 0.011) observed at the participation level, the overall WHODAS 2.0 score did not show statistical improvement (*p* = 0.248). This suggests that healthcare professionals should consider prescribing powered mobility devices like PSs to enhance well-being and independence in various environments, even though the overall disability level remained unchanged. The utilization of motored PSs can substantially improve the lives of elderly individuals by fostering independence, encouraging social engagement, and enhancing overall well-being, all while potentially proving to be cost-effective within the broader societal context [69].

Based on the reviewed evidence, the following practical recommendations are put forth to improve the findings’ practical implications for rehabilitation service providers, policymakers, and clinicians: (1) Personalized rehabilitation plans to optimize treatment effectiveness and patient satisfaction. This approach is supported by the evidence of improved outcomes in personalized treatments. (2) Integrating of emerging technologies, particularly for remote rehabilitation. These technologies have demonstrated promise in improving rehabilitation’s effectiveness and accessibility. Clinicians and rehabilitation facilities need to be properly trained and equipped to use these technologies. (3) Longitudinal and outcome-based research should prioritize the assessment of improvements in disability levels and the sustainability of these improvements, as well as the long-term effects. (4) Telerehabilitation programs. These programs should be designed to be accessible and user-friendly for patients with varying levels of technological literacy and disability. (5) Incorporating of patient-reported outcomes. These data can provide valuable insights into the acceptability and impact of rehabilitation interventions from patients’ perspectives, informing continuous improvement of service. (6) Training and education. This will ensure that patients receive the most current and effective care. (7) Policy and funding support. Policies should support the accessibility of rehabilitation services for all individuals, regardless of location or socioeconomic status. (8) Multidisciplinary teams. These offer a holistic understanding of patient needs.

While the review encompasses a wide range of conditions, it acknowledges that certain less-studied conditions for rehabilitation approach might also benefit from rehabilitation interventions. Also, understanding cultural factors specific to different cultures is essential for implementing rehabilitation services in a culturally appropriate manner and to enhance our ability to address the social dimension of disability. Future research could focus into the effects of rehabilitation on these conditions, including sarcopenia, inflammatory bowel disease, and rheumatic conditions like scleroderma. Additionally, the continuous emergence of new technologies presents an opportunity for rehabilitation. Future studies should examine the effectiveness and accessibility of these technologies for rehabilitation purposes, especially for remote or underserved populations. This review indicates that although various interventions appear promising in reducing disability, there is a scarcity of data concerning their long-term outcomes. Therefore, future research should prioritize longitudinal studies to evaluate the sustainability of disability improvement and the enduring impact of rehabilitation interventions on patients’ QoL. Addressing these gaps will contribute to a more comprehensive understanding of effective rehabilitation strategies, ultimately enhancing the quality of care and outcomes for individuals with disabilities.

## 5. Conclusions

In conclusion, there is a wide range of rehabilitation interventions, varying from CnvT to VR, RAT, exergaming, and telerehabilitation, that effectively reduce disability across various health conditions, such as those of the cardiovascular system (stroke, CVD, CHF), pulmonary system (COPD), neurologic system (PD, CP), and musculoskeletal system (orthopaedic surgery, NDs), cancer, and chronic pain, and among frail elderly patients, as evidenced by series evaluations with the WHODAS 2.0 questionnaire. Furthermore, WHODAS 2.0 is helpful for evaluating the general population’s health and disability levels through surveys, as well as for estimating the productivity gains and clinical efficacy of interventions, but WHODAS 2.0 offers the best results if used in combination with other evaluation methods. While new technologies have gained increasing prominence in recent times, CnvT remains a fundamental component of rehabilitation, offering a traditional approach to patient care. Adopting a personalized and patient-centred care strategy is essential for global health systems to enhance QoL and reduce the economic impact of disability. This emphasises the global applicability of these interventions, the shift towards personalized treatment, and the significance of considering patient needs and preferences.

## Figures and Tables

**Table 1:** Newcastle–Ottawa Scale analysis of the included articles.

Author (Reference)	Selection				Comparability	Outcome			Total Score	Quality
	Representativeness of the Exposed Cohort	Selection of the Non-Exposed Cohort	Ascertainment of Exposure	Demonstration that Outcome of Interest Was Not Present at Start of Study	Comparability of Cohorts on the Basis of the Design or Analysis	Assessment of Outcome	Was Follow-Up Long Enough for Outcomes to Occur	Adequacy of Follow-Up of Cohorts		
Dabrowska et al. [21], 2023	*	*	*	-	*	*	-	*	6	good
Fukuta et al. [22], 2023	*	*	*	*	*	*	-	*	7	good
Rossetto et al. [23], 2023	*	-	*	*	-	*	-	*	5	fair
Kilkki et al. [24], 2023	*	*	*	*	*	*	-	*	7	good
McAndrew et al. [25], 2022	*	-	*	*	*	*	-	*	6	good
Jen et al. [26], 2021	*	-	*	*	-	*	*	*	6	good
Chen et al. [27], 2021	*	*	*	*	*	*	*	*	8	good
Campbell et al. [28], 2019	*	-	*	*	-	*	*	*	6	good
Nguyen et al. [29], 2021	*	-	*	*	-	*	*	*	6	good
MacDonald et al. [30], 2020	*	-	*	*	-	*	*	*	6	good
Hustoft et al. [31], 2019	*	-	*	*	-	*	*	*	6	good
Ferraz et al. [32], 2018	*	-	*	*	-	*	*	*	6	good
Ya and Petrini [33], 2017	*	*	*	*	-	*	*	*	7	good
Galli et al. [34], 2018	*	*	*	*	-	*	*	*	7	good
Teixeira-Machado et al. [35], 2017	*	*	*	*	-	*	*	*	7	good
Shahbazi et al. [36], 2016	*	*	*	*	*	*	*	*	8	good
Petterson et al. [37], 2015	*	-	*	*	-	*	*	*	6	good
Martinez et al. [38], 2014	*	*	*	*	*	*	*	*	8	good

“*” indicate that the article meets the criteria mentioned above; “-” indicate that the article does not meet the criteria mentioned above.

**Table 2:** Study characteristics—the first author, year of publication, geographic region, WHODAS 2.0 version used (12-item, 36-item), study design, sample size, average age of participants, and condition that led to disability.

Author (Reference)	Country	WHODAS 2.0 Items	Type of Study	Number of Patients	Mean Age in Years (SD)	The Condition That Led to Disability
Dabrowska et al. [21], 2023	Czech Republic	36 items, interview	RCT	50	IG 59.36; CG 62.96	Ischaemic stroke in the arteria cerebri media
Fukuta et al. [22], 2023	Japan	12 items	Quasi-RCT	31	IG 72.2 (10.4); CG 77.3 (4.8)	CVD
Rossetto et al. [23], 2023	Italy	36 items	Usability and acceptability study—user experience research	112	70.6	CVD CHF PD COPD
Kilkki et al. [24], 2023	Finland	12 items (self and proxy)	Longitudinal cohort study	65	62.7	Stroke (ischemic, ICH, SAH)
McAndrew et al. [25], 2022	United States	12 items at screening, 36 items at 4, 12 weeks and 6 months	Multicentre RCT	268	52.9 (7.3)	Gulf War illness and disability
Jen et al. [26], 2021	Taiwan	36 items	Longitudinal follow-up study	3506	62.2 (12.5)	Stroke
Chen et al. [27], 2021	China	36 items	Assessor-blinded, prospective, pilot RCT	20	IG 46.2 (7); CG 48.6 (9.95)	Right-hemisphere stroke with left-sided unilateral spatial neglect
Campbell et al. [28], 2019	Finland	12 items, self-completed	Mixed methods	4	43.25 (11.03)	Chronic pain and potential comorbid depressive and anxious symptoms
Nguyen et al. [29], 2021	Vietnam	12 items	Cross-sectional	951	Not reported	Stroke and comorbid conditions
MacDonald et al. [30], 2020	Canada	12 items	Mixed-method pilot study	35	55 (15.9)	Cancer
Hustoft et al. [31], 2019	Norway	36 items	Longitudinal survey-based design	701	Not reported	Cancer, disease of the nervous system/musculoskeletal system/circulatory system
Ferraz et al. [32], 2018	Brazil	36 items	Randomized, controlled, single-blinded	62	69 (5)	Elderly patients with PD
Ya and Petrini [33], 2017	China	36 items	Quasi-experimental nonrandomized trial	24	IG 48.69 (11.10); CG 47.73 (10.82)	Polio, traumatic injury, born with disability, CP, spastic paraplegia
Galli et al. [34], 2018	Italy	36 items, 12-item version	Prospective multicentre observational study	80 (48 hip prostheses, 32 knee prostheses)	70.1 (1.067)	Elective hip or knee arthroplasty
Teixeira-Machado et al. [35], 2017	Brazil	12 items	RCT	26	CG 18 (3.46); IG 17.07 (2.36)	CP
Shahbazi et al. [36], 2016	Iran	36 items	Case control	92 (46 + 46)	68.53 (6.1)	Chronic disease, older people, frailty
Petterson et al. [37], 2015	Sweden	36 items	Prospective study	45	Men 79.1 (5.76); women 74.3 (7.11)	Old individuals with mobility limitations
Martinez et al. [38], 2014	Spain	36 items	Quasi-experimental study with a pre-test and post-test design	45 (21 CG, 24 EG)	Not reported	NDs MG, FSHD, BMD, LGMD

RCT—randomized controlled trial; IG—interventional group; CG—control group; CVD—cardiovascular disease; CHF—chronic heart failure; PD—Parkinson’s disease; COPD—chronic obstructive pulmonary disease; CP—cerebral palsy; ICH—intracranial haemorrhage; SAH—subarachnoid haemorrhage; NDs—neuromuscular diseases; MG—myasthenia gravis; FSHD—facioscapulohumeral muscular dystrophy; BMD—Becker muscular dystrophy; LGMD—limb–girdle muscular dystrophy.

**Table 3:** Study characteristics—the first author, year of publication, rehabilitation intervention details, values of WHODAS 2.0 before and after the intervention, and other evaluation tools.

Author (Reference)	Rehabilitation Intervention	Time of Evaluation	WHODAS 2.0 IG	WHODAS 2.0 CG	WHODAS 2.0	*p*	Evaluation Tools
Dabrowska et al. [21], 2023	IG = VR + CnvT CG = CnvT	Baseline	31.5 (18–62.6)	27.4 (17.2–36.8)	NR	0.261	MMSE BI EBI BBS WHODAS 2.0 FAC
		After the intervention	22.6 (11.3–50.7)	21.6 (15.3–30.6)	NR	0.740	
		4 weeks after the intervention	20.1 (9.2–43.8)	20.6 (15.1–29.2)	NR	0.996	
Fukuta et al. [22], 2023	IG = remote CR for 12 weeks CG = hospital-based CR for 12 weeks	Baseline	21.6 ± 14.3	19.3 ± 21.8	NR	0.06	WHOQOL-BREF WHODAS 2.0
		CG baseline vs. 12 weeks after therapy	NR	NR	12.3 ± 7.4	0.31	
		IG baseline vs. 12 weeks after therapy	NR	NR	21.5 ± 23	<0.05	
Rossetto et al. [23], 2023	Telerehabilitation program—SIDERA^B	Baseline (PD, COPD, CHF)	NR	NR	15.95	0.346	WHODAS 2.0 SUS TAM3 SUTAQ
Kilkki et al. [24], 2023	Rehabilitation, self-perceived	Discharge Md (IQR)			15.0 (14)	0.004	WHODAS 2.0 mRS FIM
		9–50 month Md (IQR)			9.00 (18)		
	Rehabilitation, proxy-perceived	Discharge Md (IQR)			20.0 (22)	<0.00001	
		9–50 month Md (IQR)			10.0 (21)		
McAndrew et al. [25], 2022	IG = PST CG = HE	Baseline mean (SE)	46.7 (1.9)	45.1 (1.9)		NR	WHODAS 2.0 Problem-solving inventory 3-item pain scale Pain Disability Index Fatigue Severity Scale
		4 weeks	42.5 (2.0)	44.6 (2.0)		NR	
		12 weeks	43.9 (2.0)	42.8 (2.0)		0.78	
		6 months	44.1 (2.2)	46.2 (2.1)		0.04	
Jen et al. [26], 2021	Existing Taiwanese healthcare system services	Baseline			49.8	<0.05	WHODAS 2.0
		4 years			47.3		
Chen et al. [27], 2021	IG = RAT CG = CnvT	Baseline (SD)	122.10 (10.84)	124 (11.43)		0.71	MoCA BIT-C CBS FMA-UE MBI WHODAS 2.0
		4 weeks (SD)	98.60 (8.70)	107.80 (11.70)		NR	
Campbell et al. [28], 2019	Vibroacoustic treatment Self-care treatment	Phase I beginning			P1-17		WHODAS 2.0 VAS Beck’s Depression Inventory-II Hospital Anxiety and Depression Scale
					P2-22		
					P3-25		
					P4-11		
		Phase I ending			P1-17		
					P2-20		
					P3-12		
					P4-8		
		Phase III beginning			P1-17		
					P2-28		
					P3-9		
					P4-9		
		Phase III ending			P1-18		
					P2-23		
					P3-5		
					P4-5		
		Follow-up			P1-19		
					P2-28		
					P3-10		
					P4-9		
Nguyen et al. [29], 2021	IG = Diet-quality modifications CG = PA				32.3 (13.5)		Physical Activity Questionnaire, short version DASH-Q IPAQ-SF CCI
MacDonald et al. [30], 2020	CaRE@Home intervention	Baseline	9.84 (1.14)			NR	WHODAS 2.0 GSLTPAQ iMTA PCQ 6 MWT Grip strength BMI Resting heart rate Blood pressure
		8-weeks post-intervention	8.17 (1.01)			0.03	
		3 months post-intervention	7.56 (1.10)			0.008	
Hustoft et al. [31], 2019	3-week rehabilitation process	Baseline			28.6 (15.9)	NR	WHODAS 2.0 EQ-VAS NCQ-N
		1 year			24.1 (15.9)		
Ferraz et al. [32], 2018	Functional training	Baseline			73.3 (22.0)	0.018	6MWT 10MWT SRT BMI PDQ-39 WHODAS 2.0 15-item Geriatric Depression Scale
		8 weeks			63.91 (14.0)		
	Bicycle exercise	Baseline			66.2 (17.7)	0.019	
		8 weeks			61.9 (16.2)		
	Exergaming with Xbox 360 video game *Kinect*	Baseline			70.75 (19.6)	0.041	
		8 weeks			64.3 (19.2)		
Ya and Petrini [33], 2017	Home-based resistance exercise	Baseline			19.30 (13.58)	0.007	6MWT FTSST 10MWT TUG One-repetition maximum, WHODAS 2.0 SF-36 1RM
		12 weeks			11.19 (8.23)		
	Encouraged to do more exercise	Baseline			11.42 (12.31)	0.848	
		12 weeks			11.77 (12.40)		
Galli et al. [34], 2018	Inpatients rehabilitation	Baseline			37.28 (9.070)		ADL BI WHODAS 2.0
		30 days			15.65 (11.658)	0.002	
Teixeira-Machado et al. [35], 2017	IG- = dance class sessions CG = kinesiotherapy	Baseline	84.56 (%)	84.45 (%)		0.33	WHODAS 2.0 FIM GMFCS
		After 24 sessions	39.90 (%)	69.55 (%)		0.04	
Shahbazi et al. [36], 2016	IG—care service package CG—potential candidates for receiving care service package	Baseline	23.08 (12.16)	21.98 (11.55)		0.659	WHODAS 2.0
		Before treatment	22.6 (11.2)	22.0 (11.5)			
		2 months after treatment	19.3 (10.6)	22.6 (11.0)			
		4 months after treatment	17.6 (9.3)	24.7 (11.1)			
		6 months after treatment	17.4 (8.9)	25.8 (10.8)		<0.001	
Petterson et al. [37], 2015	IG—4 months after PMDs CG—before PMD		24.57 (12.16)	28.12 (13.20)		0.248	WHODAS 2.0 IPPA SF-36
Martinez et al. [38], 2014	IG—online intervention CG—no intervention	Pre-intervention	34.71 (18.96)	14.47 (12.38)		0.002	WHODAS SIP SF-36
		Post-intervention	25 (17.79)	16.3 (14.55)		0.180	

WHODAS 2.0—WHO Disability Assessment Schedule 2.0; IG—interventional group; CG—control group; VR—virtual reality; CnvT—conventional therapy; NR—not reported; MMSE—minimal mental state examination; BI—Barthel Index; EBI—Extended BI; BBS—Berg Balance Scale; FAC—Functional Ambulatory Category; CR—cardiac rehabilitation; WHOQOL-BREF—shortened version of the World Health Organization (WHO) quality of life scale; PD—Parkinson’s disease; COPD—chronic obstructive pulmonary disease; CHF—chronic heart failure; SUS—System Usability Scale; TAM3—Technology Acceptance Model 3; SUTAQ—Service User Technology Acceptance Model 3; Md—median; IQR—interquartile range; mRS—modified Rankin Scale; FIM—functional independence measure; PST—problem-solving treatment; HE—health education; SE—standard error; MoCA—Montreal Cognitive Assessment; BIT-C—Behavioural Inattention Test—conventional section; CBS—Catherine Bergego Scale; FMA-UE—Fugl-Meyer Assessment for Upper Extremity; MBI—modified Barthel Index; RAT—robot-assisted arm training; P—patient; VAS—Visual Analogue Scale; PA—physical activity; DASH-Q—Dietary Approaches to Stop Hypertension Quality questionnaire; CCI—Charlson Comorbidity Index; PDQ-39—Parkinson’s Disease Questionnaire-39; IPAQ-SF—International Physical Activity Questionnaire, short version; GSLTPAQ—Godin–Shephard Leisure-Time Physical Activity Questionnaire; iMTA PCQ—Productivity Cost Questionnaire; BMI—body mass index; EQ-VAS—EuroQol VAS; NCQ-N—Norwegian version of the Nijmegen Continuity Questionnaire; SRT—sitting–rising test; 6MWT—6 min walk test; FTSST—Five-Time Sit-to-Stand Test; 10MWT—10 m walk test; TUG—Timed Up and Go; 1RM—one-repetition maximum; SF-36—Self-administered Short Form Health Survey; ADL BI—activities of daily living modified scale; GMFCS—Gross Motor Function Classification System; PMDs—powered mobility devices; IPPA—Individually Prioritized Problem Assessment; SIP—Sickness Impact Profile.

## Data Availability

The original contributions presented in the study are included in the article, further inquiries can be directed to the corresponding authors.
